# Challenges faced by parents of screen-detected children with Cystic Fibrosis: The ICOS study

**DOI:** 10.1093/eurpub/ckac131.214

**Published:** 2022-10-25

**Authors:** R Bhatnagar, B Linnane, M Herzig, M Ni Chroinin, D Cox, B Elnazir, R Segurado, L Kirwan, KW Southern, P Fitzpatrick

**Affiliations:** 1 School of Public Health, Physiotherapy and Sports Science, University College Dublin, Dublin, Ireland; 2 University Hospital Limerick, Limerick, Ireland; 3 University Hospital Galway, Galway, Ireland; 4 Cork University Hospital, Cork, Ireland; 5 Children’s Health Ireland at Crumlin, Dublin, Ireland; 6 Children’s Health Ireland at Tallaght, Dublin, Ireland; 7 Trinity College Dublin, Dublin, Ireland; 8 Cystic Fibrosis Registry, Dublin, Ireland; 9 University of Liverpool, Liverpool, UK

## Abstract

**Background:**

Informal care is an essential component of overall care for patients, particularly those with chronic illnesses such as Cystic Fibrosis (CF). This study aims to assess the level of caregiving burden faced by parents/caregivers of children with CF (CwCF) recruited to the Irish Comparative Outcomes Study of CF (ICOS), a historical cohort study of CwCF. In July 2011, a new-born screening programme began in Ireland.

**Methods:**

The study population includes the parents of screen-detected CwCF born between July 2011-2021. The Challenge of Living with CF-Short Form is a new, validated 15-item tool that evaluates the caregiving burden faced by parents from the child’s diagnosis until early adolescence. Comparisons based on the age of screen-detected CwCF were conducted. SPSS was used for analysis.

**Results:**

69 parents of screen-detected CwCF responded. Fifty percent of parents of older children (aged 4-12+ years) and 35% of the parents of toddlers (0-3 years) faced moderate-high level difficulties in managing the extra expenses required for the care of their CwCF, despite all children receiving free clinical care, prescriptions and medications. A significantly greater proportion of the parents of older children than younger children experienced constant problems in managing daily oral medication routines (37% vs 13%; P = 0.039), nebulised medication routines (67.5% vs 21.4%; P = 0.003), and physiotherapy routines (57.8% vs 31.8%; P = 0.046)

**Conclusions:**

Using the novel Challenge of living with Cystic Fibrosis-Short Form questionnaire, our findings suggest that the caregiving burden is higher for parents of older CwCF. Expenses incurred by parents of a child with a serious chronic medical condition go beyond medical care and treatment expenses.

**Key messages:**

• The challenge of living with Cystic Fibrosis-Short Form is being used for the first time in a population setting.

• The caregiving burden was more pronounced in the parents of older CwCF.

